# Deep Learning Enables Optofluidic Zoom System with Large Zoom Ratio and High Imaging Resolution

**DOI:** 10.3390/s23063204

**Published:** 2023-03-17

**Authors:** Jiancheng Xu, Fenglin Kuang, Shubin Liu, Lei Li

**Affiliations:** School of Electronics and Information Engineering, Sichuan University, Chengdu 610065, China

**Keywords:** electrowetting liquid lens, deep learning, optofluidic zoom system

## Abstract

Due to the relatively low optical power of a liquid lens, it is usually difficult to achieve a large zoom ratio and a high-resolution image simultaneously in an optofluidic zoom imaging system. We propose an electronically controlled optofluidic zoom imaging system combined with deep learning, which achieves a large continuous zoom change and a high-resolution image. The zoom system consists of an optofluidic zoom objective and an image-processing module. The proposed zoom system can achieve a large tunable focal length range from 4.0 mm to 31.3 mm. In the focal length range of 9.4 mm to 18.8 mm, the system can dynamically correct the aberrations by six electrowetting liquid lenses to ensure the image quality. In the focal length range of 4.0–9.4 mm and 18.8–31.3 mm, the optical power of a liquid lens is mainly used to enlarge the zoom ratio, and deep learning enables the proposed zoom system with improved image quality. The zoom ratio of the system reaches 7.8×, and the maximum field of view of the system can reach ~29°. The proposed zoom system has potential applications in camera, telescope and so on.

## 1. Introduction

Traditional zoom imaging devices are usually large in overall size and take a long time to change the focal length by mechanical movement [1,2,3,4,5,6]. To achieve a lightweight, fast response and no mechanical disturbance of the zoom system, liquid lenses provide a solution; liquid lenses vary focal length by changing the radius of the liquid–liquid (L–L) surface. Among the liquid lenses, the electrowetting liquid lens is one of the ideal ways due to fast response speed, no gravity effect and good imaging quality. Optical zoom systems based on an electrowetting liquid lens have become a research hotspot in the field of optics. Many zoom systems based on electrowetting liquid lenses have been proposed [7,8,9,10,11,12,13]. For example, a two-liquid-lenses zoom system was proposed, in which a zoom factor of 1.8 was implemented [7]. Another electrically optofluidic zoom system that can achieve a continuous zoom change and high-resolution image and the zoom ratio is over 2.6× [11]. In addition, an all-liquid optical zoom system based on electrowetting theory with a 1.5× zoom ratio was proposed [10]. The lenses are individually and independently tunable using electrowetting, allowing a flexible reconfiguration of the zoom. However, the limited aperture of the electrowetting liquid lens makes it difficult to balance high resolution and a large zoom ratio. In general, the larger the zoom ratio, the more difficult the aberration correction is. The limitations of liquid materials (low refractive index, small Abbe number) also necessarily bring corresponding aberration problems to optical systems. At this time, it is difficult to correct the chromatic aberration in some extreme cases. In general, the electrowetting liquid lens can be used to correct aberration with its variable optical power, but the limited optical power range affects the ability for correcting aberration. In addition to optimizing the optical structure, some image-processing algorithms were proposed to improve the image quality [14,15,16,17,18,19,20,21]. For instance, a real-time digital correction method for transverse chromatic aberration was presented, which can be adapted to multi-resolution foveated laparoscope systems [14]. A hybrid refractive-diffractive lens was proposed to be paired with a deep neural-network-based image reconstruction. In addition, it produces high-quality, real-world images with minimal artifacts, reaching a PSNR of 28 dB on the test set [19]. Moreover, an approach based on a generative adversarial network was proposed to simultaneously denoise and super-resolve OCT images [15]. Usually, the image processing is time-consuming, and it is difficult to use in a real-time zoom system.

In this paper, we propose and demonstrate an optofluidic zoom imaging system that can achieve an adjustable focal length range of 4.0 mm to 31.3 mm and a zoom ratio of 7.8×. The zoom system is designed based on the electrowetting liquid lens combined with the idea of deep learning image processing. To obtain a large zoom ratio and high imaging resolution, we divided the focal length into three parts for optimization. In the focal length range of 9.4 mm to 18.8 mm, the system can dynamically correct the aberrations by the electrowetting liquid lenses to ensure the image quality. In the focal length range of 4.0–9.4 mm and 18.8–31.3 mm, super-resolution (SR) and aberration correction algorithms are used to improve the image quality and achieve the purpose of large zoom ratio and high resolution. The system combines the fast zoom of an electrowetting liquid lens with the advantages of the algorithm in image-quality improvement processing, and reduces the influence of the small optical power range of an electrowetting liquid lens and slow algorithm processing.

## 2. System Structure

**Figure 1:**
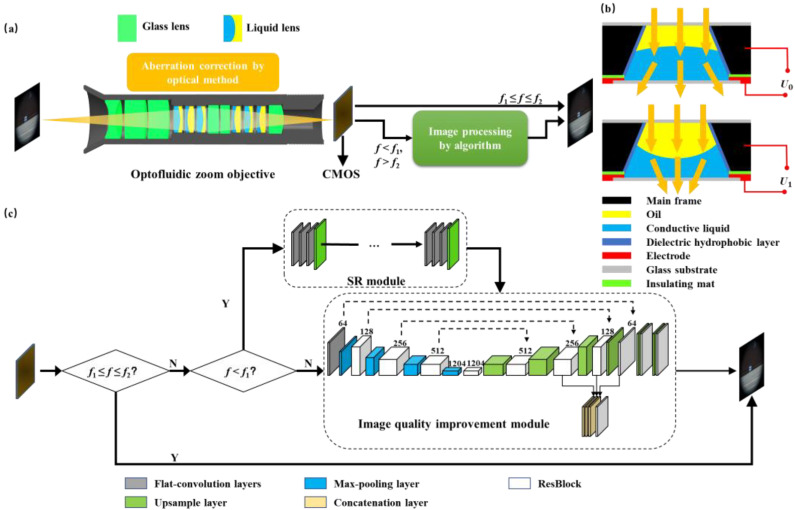
Schematic cross-sectional structure and the principle of the proposed zoom system. (**a**) Structure of the proposed zoom system. (**b**) Structure and principle of the electrowetting lens. *U*_1_ and *U*_0_ are different applied voltages. The yellow arrows represent the direction of light propagation. (**c**) Flow chart of image processing.

The proposed zoom system is shown in Figure 1. It consists of two main parts: an optofluidic zoom objective and an image-processing module.

### 2.1. Optofluidic Zoom Objective

As the core component, the optofluidic zoom objective is specially designed and manufactured to achieve continuous optical zoom with a large zoom ratio. As shown in Figure 1a, the optofluidic zoom objective is composed of six liquid lenses and glass lenses, and its focal length can be changed by changing the interface of the electrowetting liquid lens. The used liquid lens is an electrowetting-actuated lens with variable focal length. The structure of the electrowetting lens is shown in Figure 1b. The main frame and two glass substrates are placed together to form a chamber. The main frame is made of aluminum, which serves as an electrode. The inner wall of the main frame is coated with a dielectric hydrophobic layer. The conductive liquid and the oil form a liquid–liquid interface, which can be tuned by applied voltage. Its focal length is changed due to an electrowetting effect. According to the Young–Lippmann equation, the relationship of the contact angle θ and the applied voltage U can be described as follows [22]:

(1) $$cos\theta =\frac{\gamma_{1}-\gamma_{2}}{\gamma_{12}}+\frac{\varepsilon }{2\gamma_{12}d}U^{2},\;$$

where ε is dielectric constant of the insulating layer, d is the thickness of the insulating layer, and $\gamma_{1}$, $\gamma_{2}\;$ and $\gamma_{12}\;$ are the interfacial tensions of sidewall/oil, sidewall/water and oil/water, respectively.

The optofluidic zoom objective can dynamically correct aberrations within a limited focal length range. However, due to aperture limitations, it is difficult to maintain a large zoom ratio and high resolution at the same time. For the proposed zoom system, when the focal length *f*_1_ ≤ *f* ≤ *f*_2_ (*f*_1_ = 9.4 mm, *f*_2_ = 18.8 mm), the aberration is corrected by six liquid lenses. However, when the focal length *f* ≤ *f*_1_ or the focal length *f* ≥ *f*_2_, the main power of the liquid lenses is used to enlarge the zoom ratio, and thus it is very difficult to correct aberration effectively. Therefore, the deep learning method is used to improve image quality.

### 2.2. Image Processing Module

The flow chart of image processing is shown in Figure 1c, including a super-resolution module to improve resolution and an image-quality improvement module to correct image defects. The limit of the aperture of the electrowetting liquid lens will seriously block the FOV (Field of View) of the system, which will reduce the imaging range. If the image resolution is low, the effect after processing by the deep learning algorithm is not significant. So we apply a super-resolution algorithm to improve image resolution for image enhancement. The super-resolution module consists of *N* identical blocks, each of which includes three convolution layers and one upsampling layer. The image with low resolution is used as the input of the super-resolution module. Three convolution layers are used for feature extraction, then the upsample layer is used to enlarge the image size, and the process is repeated N times to increase the image resolution. The image-quality improvement module is composed of an encoder–decoder network. The encoder is composed of a flat-convolution layer, several max-pooling layers and residual blocks (Resblock). The maximum pooling layer is used to compress the features extracted from the convolution layer. At the same time, it can simplify the network complexity and improve the computing speed. The decoder is composed of several upsampling layers and concatenation layers. The Resblocks and the concatenation layers can contribute to the rapid convergence of the network, thus producing a clearer output. At the end of the network, there are two upsampling layers to restore image resolution. Set5 [23] is used as a dataset for training. The loss function of the network is:

(2) $$MSE=\frac{1}{n}\sum_{i=1}^{n}{\left(f\left(x\right)-y\right)}^{2},\;$$

where n is the number of samples, $f\left(x\right)$ is the predicted value, and y is the true value. In this way, the SR module and the image-quality improvement module are combined to solve the problem of resolution reduction caused by the uncorrectable aberration and the limit of the aperture of the electrowetting liquid lens.

The combination of optical correction and image processing maximizes the potential of the electrowetting liquid lenses to achieve a large zoom ratio and high resolution while ensuring efficiency.

## 3. Design and Fabrication

According to the parameters of the initial structure, we simulated the optical structure in the 4.0–31.3 mm focal length range in Zemax and optimized the imaging quality. We set the radii $\left(r_{1},r_{2},r_{3},r_{4},r_{5},r_{6}\right)$ of the six electrowetting liquid lenses as variables in the Multiple Configuration Editor. To obtain the required focal length, the operands controlling the focal length were added to the merit function in Zemax. The merit function is constructed based on Equations (3)–(5).

(3) $$f_{0}=F_{1}\left(r_{1},r_{2},r_{3},r_{4},r_{5},r_{6}\right)$$

(4) $$L_{0}=F_{2}\left(r_{1},r_{2},r_{3},r_{4},r_{5},r_{6}\right)$$

(5) $$RMS=F_{3}\left(r_{1},r_{2},r_{3},r_{4},r_{5},r_{6}\right)$$

where $f_{0}$ is the required focal length, and $L_{0}$ is the back focal distance (fixed). RMS is the root mean square of the ray traced blur spots, and the target value is 0. $F_{1}\left(r_{1},r_{2},r_{3},r_{4},r_{5},r_{6}\right)$ and $F_{2}\left(r_{1},r_{2},r_{3},r_{4},r_{5},r_{6}\right)$ are calculated based on the ray trace method [11]. $F_{3}\left(r_{1},r_{2},r_{3},r_{4},r_{5},r_{6}\right)$ is included in the Default Merit Function in Zemax. We set the weights of short focus and long focus higher to maximize the zoom ratio.

**Figure 2:**
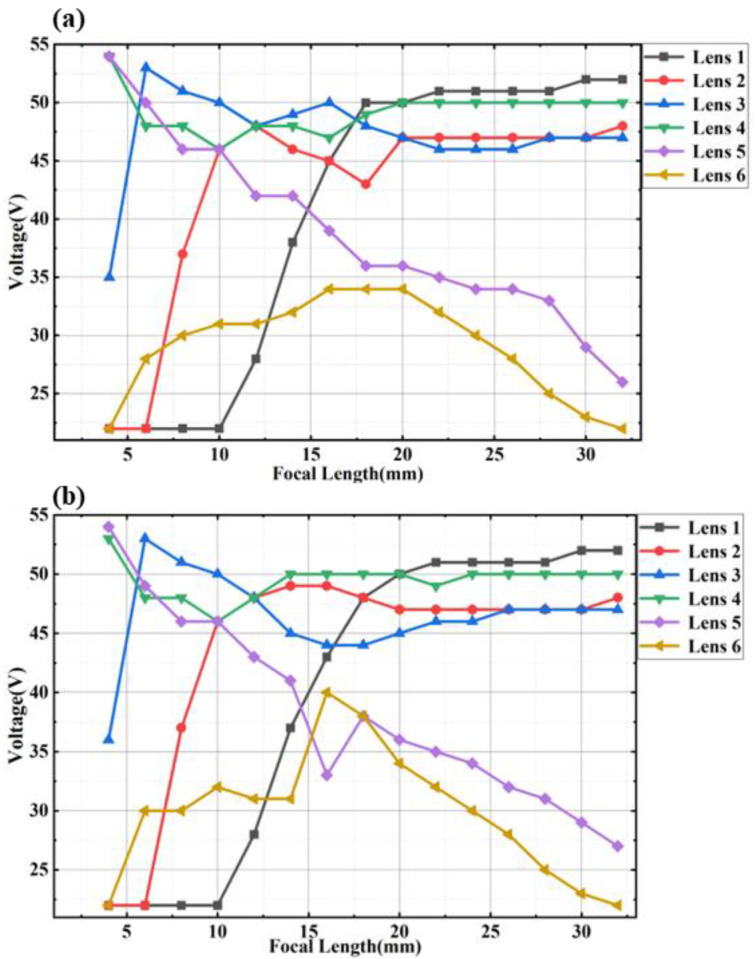
Focal length of the system versus voltages. (**a**) Zemax simulation data. (**b**) Experimental data.

After optimization, we obtained the optimized radii of six electrowetting liquid lenses at each focal length and converted the radii into voltages. Then the optimized voltages were applied to the six electrowetting liquid lenses. The radii of curvature of liquid lenses and glass lenses and materials of glass lenses are shown in Table 1. Because of fabrication error, we usually need to slightly adjust the voltage of one of the electrowetting liquid lenses until the image is clearest. In addition, the change of ambient temperature will also lead to errors between the actual system and the ideal structure, which causes the voltage applied in the experiment to slightly deviate from the simulation. Figure 2 shows several solutions in the focal range and the corresponding voltages. Compared with Figure 2a,b, the images show that the distribution is similar in the overall trend and slightly different, which shows that the results of the experiment and simulation are roughly the same. For example, when the focal length is 16 mm, the difference between the experimental and simulation is large. However, the optical powers of Lens 1, Lens 3 and Lens 6 become smaller (the voltage is greater than 40 V, which is positive), and the optical powers of Lens 2, Lens 4 and Lens 5 become larger accordingly. The six liquid lenses compensate each other to zoom.

**Figure 3:**
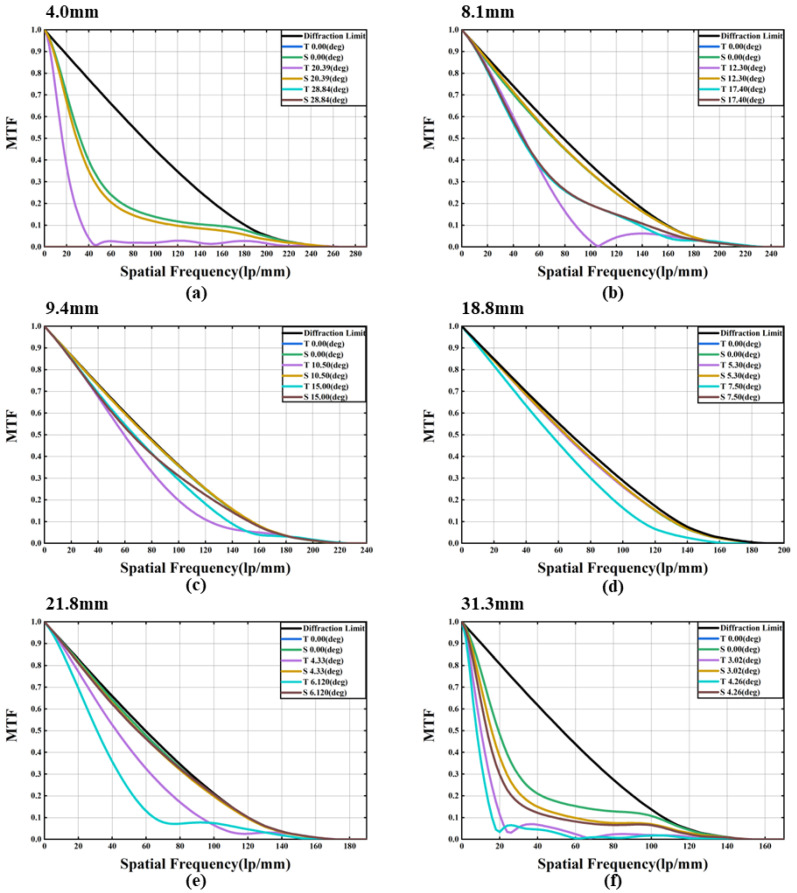
MTF of the proposed zoom system in the whole tuning range. “T” represents the tangential direction, and “S” represents the sagittal direction. (**a**) *f* = 4.0 mm. (**b**) *f* = 8.1 mm. (**c**) *f* = 9.4 mm. (**d**) *f* = 18.8 mm. (**e**) *f* = 21.8 mm. (**f**) *f* = 31.3 mm.

However, with the extended focal length, the imaging quality of the proposed system has also decreased significantly. The corresponding MTF (Modulation Transfer Function) is shown in Figure 3. We see that the MTF curve in the middle focal length range (9.4–18.8 mm) is close to the diffraction limit, which indicates that the aberration is corrected in the tuning range. However, we also see that not all of the imaging quality in the focal length range is very good. For example, for a large FOV (Field of View) at *f* = 4.0 mm, the MTF value drops sharply, indicating that the imaging quality rapidly deteriorates. The main reason is that the variable optical power of the liquid lens is mainly used for the zoom of the system, and the aberration cannot be corrected well.

**Figure 4:**
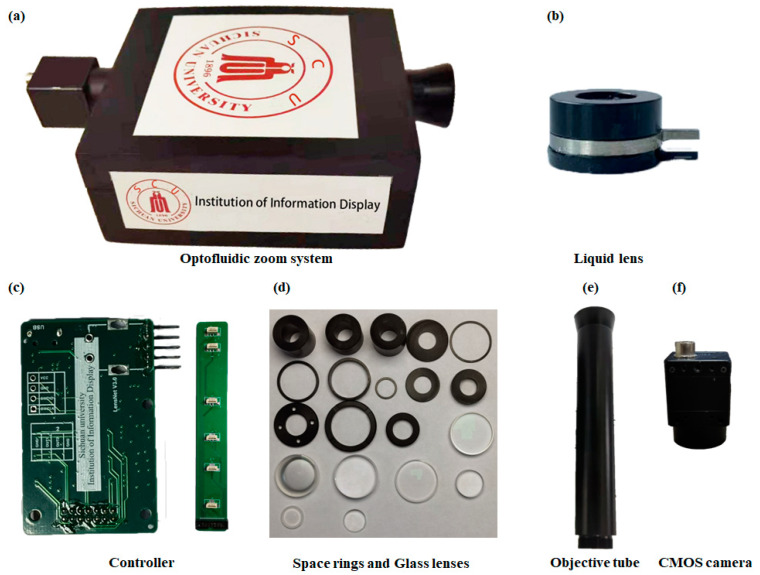
Fabricated device. (**a**) Optofluidic zoom system. (**b**) Liquid lens. (**c**) Controller. (**d**) Space rings and glass lenses. (**e**) Objective tube. (**f**) CMOS camera.

The proposed optofluidic zoom system is manufactured as shown in Figure 4a. All the fabricated elements are shown in Figure 4b–f. The fabricated electrowetting lens is shown in Figure 4b. The main frame is aluminum. Dielectric hydrophobic layer is Parylene-C followed by Teflon (AF-1600). The thickness is ~3 μm. The glass substrate is BK7. The effective aperture is ~6.2 mm. The main materials of the electrowetting liquid lenses are an oil (IOTA704, refractive index: 1.55; abbe number: 39) and a conductive liquid (refractive index: 1.40; abbe number: 45.5). The main elements consist of an optofluidic zoom objective, driving part and a CMOS camera. The CMOS is a 1/2.5″ progressive scan CMOS (ON Semiconductor Python 2000) as shown in Figure 4f. The fabricated tube is shown in Figure 4e. Its size is approximately 25 × 25 × 180 (mm). For the objective, six electrowetting liquid lenses and several glass lenses are used. The driver shown in Figure 4c is based on ARM 32-bit Cortex M3 CPU (STM32F103ZET6). It provides multiple sets of specific voltages for the system. Each set of voltage includes 6 independent voltage signals. These voltages are applied to the liquid lenses in the objective. The proposed image-quality improvement module is trained and tested on a PC (Intel Core i9-10885H CPU @2.40 GHz + RTX2080 Super) equipped with the Windows10 operating system and is based on the platform of PyTorch 1.7.0.

## 4. Experiment

**Figure 5:**
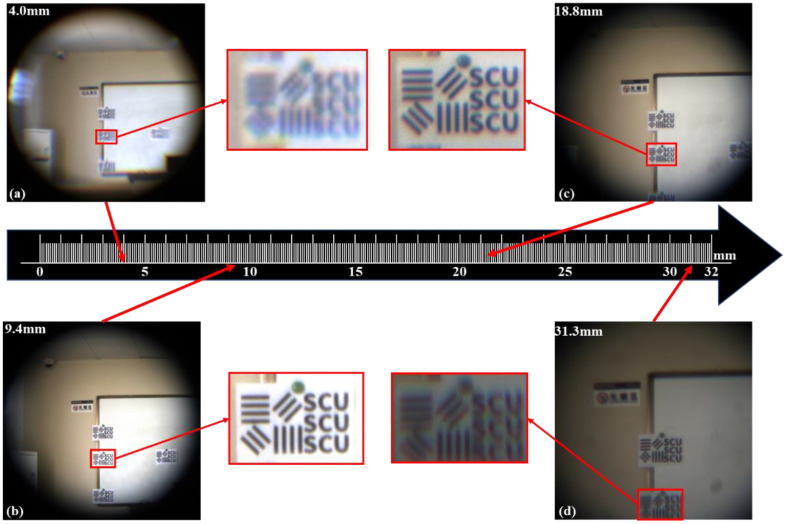
Images taken at different focal lengths. (**a**) *f* = 4.0 mm. (**b**) *f* = 9.4 mm. (**c**) *f* = 18.8 mm. (**d**) *f* = 31.3 mm.

**Figure 6:**
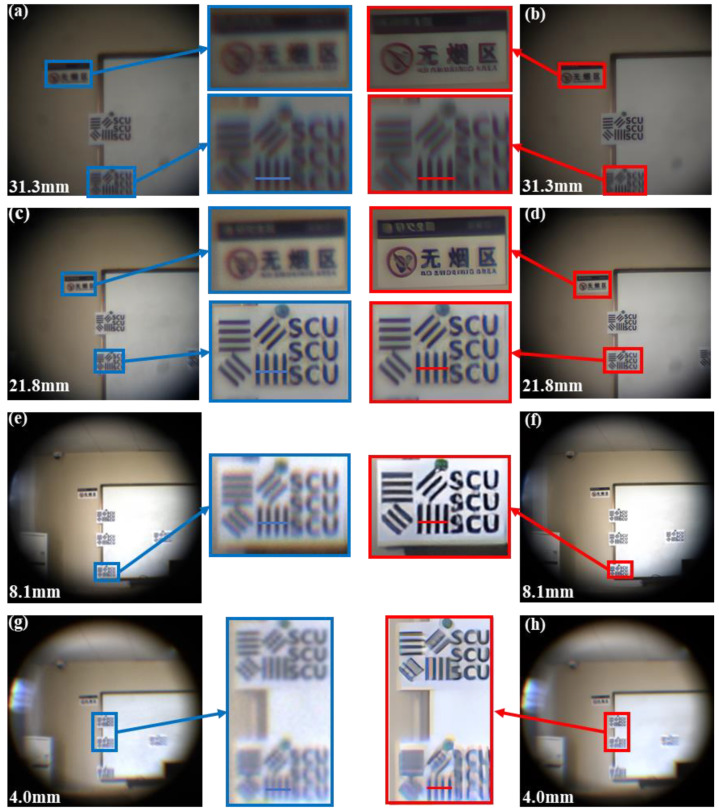
Comparison of image quality of resolution targets. (**a**) Image taken at *f* = 31.3 mm without deep learning. (**b**) Image processed at *f* = 31.3 mm by deep learning. (**c**) Image taken at *f* = 21.8 mm without deep learning. (**d**) Image processed at *f* = 21.8 mm by deep learning. (**e**) Image taken at *f* = 8.1 mm without deep learning. (**f**) Image processed at *f* = 8.1 mm by deep learning. (**g**) Image taken at *f* = 4.0 mm without deep learning. (**h**) Image processed at *f* = 4.0 mm by deep learning.

**Figure 7:**
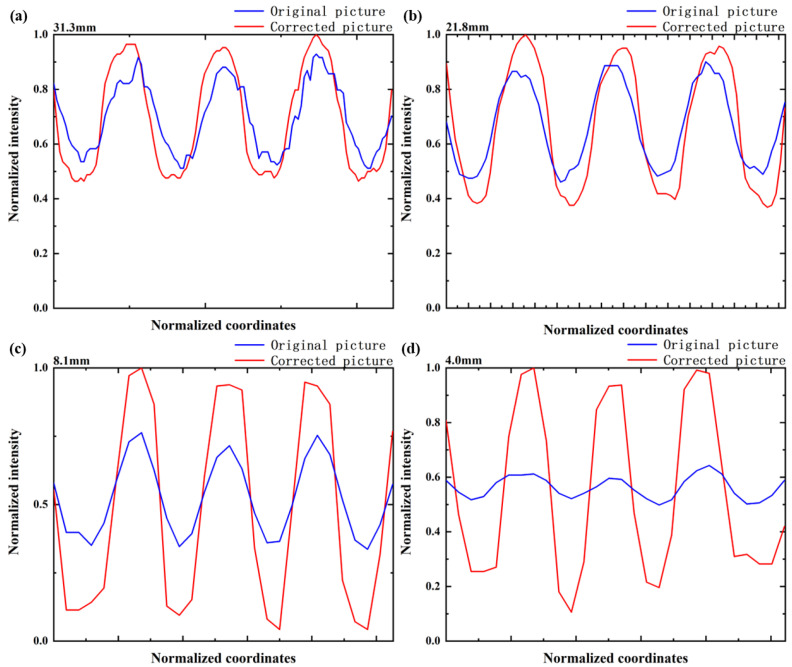
Normalized intensity before and after image processing with different focal lengths. The blue line is the normalized intensity of the original image, and the red line is the normalized intensity of the image after image processing. (**a**) *f* = 31.3 mm. (**b**) *f* = 21.8 mm. (**c**) *f* = 8.1 mm. (**d**) *f* = 4.0 mm.

To evaluate the optical performance of the proposed zoom system, we constructed a scene consisting of resolution target maps (line width is 10 mm). The target distance was ~15 m. We tested the imaging capability of this optical zoom imaging system at different focal lengths. We applied the calculated voltages to six liquid lenses to control the radii of the 6 interfaces and adjust the focal length of the system to 4.0–31.3 mm. We subsequently applied the voltages to obtain different focal lengths such as 4.0 mm, 8.1 mm, 9.4 mm, 18.8 mm, 21.8 mm and 31.3 mm, respectively. The obtained pictures are shown in Figure 5. The power of the liquid lens reaches its limit to achieve a large zoom ratio (7.8×). As shown in Figure 5, when the focal length is within the range of 9.4–18.8 mm, the imaging quality is relatively good. However, the imaging quality is significantly reduced at shorter and longer focal length (4.0–9.4 mm, 18.8–31.3 mm). To deal with the imaging quality problem at shorter and longer focal length, deep learning algorithm is used to process the imaging quality. The results are shown in Figure 6. In addition, the normalized intensity distribution before and after image processing is respectively shown in Figure 7. The contrast ratio calculation formula is as follows:

(6) $$C=\frac{I_{max}-I_{min}}{I_{max}+I_{min}}$$

where $I_{max}$ is maximum gray-scale value, $I_{min}$ is minimum gray-scale value. It can be seen that the contrast has been significantly improved after deep learning algorithm processing. Comparing the image quality in Figure 6g,h, the aberration is reduced a great deal. The quantitative comparison of the contrast ratio is shown in Table 2 and Figure 7d, where the blue line represents the intensity of the original picture and the red line represents the intensity of the picture after image processing. According to the experimental data, we calculated the contrast ratio between the original image and the corrected image, as shown in Table 2. The contrast ratio of the blue line in the center is about 0.13. The contrast ratio of the red line in the center is about 0.81. According to the experimental data, it takes about ~2 s for the SR module in the image-processing module to work once, and about ~1.5 s for the image-quality improvement module. In theory, the processing time will be shorter as the hardware level improves. The results show that the resolution has been improved by image processing. Overall, the image quality is excellent across the entire tuning range.

In order to measure the quantitative performance of the network and prove the superiority of deep learning, we calculated the peak signal-to-noise ratio (PSNR) of the deep learning. Table 3 shows the quantitative comparison between traditional image enhancement methods and deep learning. It proves that deep learning can perform better than traditional algorithms.

From the experiments, the proposed zoom system can achieve a large adjustable focal length range from 4.0 mm to 31.3 mm, together with high-resolution images. In addition, only voltages are used to zoom, without moving parts. Therefore, the proposed zoom system is very compact and easy to operate.

## 5. Conclusions

In this work, we propose an optofluidic zoom imaging system that can achieve a large continuous zoom change (from *f* = 4.0 mm to *f* = 31.3 mm). The maximum zoom ratio is 7.8× and the images are high resolution. The optofluidic zoom imaging system consists of an optofluidic zoom objective and an image-processing system. In the tuning range, the optofluidic zoom imaging system can correct aberrations and improve imaging quality by six liquid lenses and the deep learning method dynamically. The entire system is electrically controlled by using electrowetting liquid lenses. Therefore, the proposed system has no mechanical moving parts, which can greatly reduce the wear of the system and simplify the control of the system. Due to a large zoom ratio, the proposed optofluidic zoom imaging system has potential applications in cameras, telescopes and so on.

## Figures and Tables

**Table 1:** Radii of curvature of liquid lenses and glass lenses at different focal lengths and the materials of glass lenses.

**Focal length(mm)**		4.0	8.1	9.4	18.8	21.8	31.3
**Radii of curvature of liquid lenses(mm)**	#1	−7.3	−7.3	−7.3	15.1	13.6	12.1
	#2	−7.3	−25.8	14.0	24.6	25.4	22.3
	#3	−17.2	13.5	−88.9	58.2	30.4	25.8
	#4	9.6	19.1	13.5	14.1	16.1	14.0
	#5	9.9	29.4	27.1	−15.4	−17.4	−9.3
	#6	−7.3	−10.9	−10.0	−23.8	−13.0	−7.3
**Number of glass lenses**		**Radii of curvature of glass lenses(mm**)			**Material**		
#1		−225.0			N-SF8		
		125.3					
#2		175.4			N-SF8, H-LAK10		
		−570.0					
		−26.2					
#3		−40.0			H-ZF10		
		1200.0					
#4		−10.7			N-SF8		
		−55.0					
#5		9.9			H-QK1		
		−14.0					
#6		−11.2			ZF2		
		36.7					
#7		−159.3			H-FK61, H-K9L		
		−47.4					
		−17.1					

**Table 2:** Contrast ratio of the original image and the corrected image at different focal lengths.

**Focal length (mm)**	4.0	8.1	21.8	31.3
**Original image contrast ratio**	0.13	0.39	0.32	0.29
**Corrected image contrast ratio**	0.81	0.92	0.46	0.37

**Table 3:** Quantitative comparison with other traditional image-processing methods.

Method.	Histogram Equalization	Linear Contrast Adjustment	Wiener Filter	Deep Learning
**PSNR (dB)**	20.5926	23.2059	27.4773	29.6911

## Data Availability

Data available from the authors on request.
